# High relative humidity improves leaf burn resistance in flowering Chinese cabbage seedlings cultured in a closed plant factory

**DOI:** 10.7717/peerj.14325

**Published:** 2022-11-08

**Authors:** Yunyan Kang, Qiaobo Wu, Jinming Qin, Min Zhong, Xian Yang, Xirong Chai

**Affiliations:** College of Horticulture, South China Agricultural University, Guangzhou, Guangdong, China

**Keywords:** Plant factory, Leaf burn, Relative air humidity, Leaf water potential, Flowering Chinese cabbage, Light condition

## Abstract

Plant factories that ensure the annual production of vegetable crops have sparked much attention. In the present study, thirty types of common vegetable crops from 25 species and eight families, were grown in a multi-layer hydroponic system in a closed-type plant factory to evaluate the adaptive performance. A total of 20 vegetable crops, belonging to 14 species and 4 families, unexpectedly exhibited different degrees of leaf margin necrosis in lower leaves firstly, then the upper leaves gradually. We defined this new physiological disorder as “leaf burn”. It occurred more commonly and severely in cruciferous leafy vegetables. Two different light intensities (150 and 105 µmol m^−2^ s^−1^ photosynthetic photon flux density (PPFD)), three photoperiod conditions (12, 10 and 8 h d^−1^) and two canopy relative air humidity (RH) (70% and 90% RH) were set to evaluate the suppression effects on leaf burn occurrence in two commercial flowering Chinese cabbage cultivars (‘Sijiu’ and ‘Chixin’), the special cruciferous vegetable in South China. We discovered that changing light conditions did not fully suppress leaf burn occurrence in the cultivar ‘Sijiu’, though lower light intensity and shorter photoperiod partly did. Interestingly, the occurrence of leaf burn was completely restrained by an increased canopy RH from 70% to 90%. Specifically, the low RH-treated seedlings occurred varying degree of leaf burn symptoms, along with rapidly decreased water potential in leaves, while the high RH treatment significantly lessened the drop in leaf water potential, together with increased photosynthetic pigment contents, net photosynthetic rate, stomatal conductance and transpiration rate, decreased leaf stomatal aperture and density, and thus reduced the incidence of leaf burn in ‘Sijiu’ and ‘Chixin’, from 28.89% and 18.52% to zero, respectively. Taken together, high canopy RH may favor maintaining leaf water potential and improving photosynthesis performance, jointly regulating leaf burn incidence and plant growth.

## Introduction

Many crises are threatening food supply chains, including scarcity of arable land and water supplies, extreme weather conditions, geopolitical uncertainty, and epidemics, making it imperative to build a resilient food supply system. One approach to achieve this is to expand localized high-yield controlled-environment agriculture (CEA) systems, such as greenhouses, indoor vertical farms and plant factories. The scaling up of CEA could not only enable agricultural production in any weather condition or location, but also boost local consumption, shorten food transportation distances, reduce freshness loss due to long-distance transportation and storage, and cut fuel consumption (Weidner, Yang & Hamm, 2021).

Recently, plant factories with artificial lighting have attracted more and more attention since they can achieve annual production, as well as high-quality and safe production. As a closed system, it is highly airtight with minimum ventilation and high CO_2_ utilization efficiency for plant growth (Kozai, 2019). Environmental regulation is essential for plant factories, and has a notable impact on plant quality, yield, and production costs (Li et al., 2012; Villarreal-Guerrero, Pinedo-Alvarez & Flores-Velázquez, 2020). Importantly, among the many environmental regulations, humidity management is critical for optimal plant growth and development in plant factory. Regional humidity, including the absolute and relative humidity, is affected by numerous factors, such as plant transpiration rates, irrigation strategies, infiltration, ventilation, and active systems for air humidification or dehumidification. The difference between saturation and actual air vapor pressure at a given temperature is represented by vapor pressure deficit (VPD) and that it is the most accurate parameter indicating leaf water loss and has a linear relationship with plant transpiration, nevertheless, relative air humidity (RH) remains the most commonly used indicator in horticulture (Gómez et al., 2019). In the plant factory, many vegetables suffer from tip burn, a common physiological disorder characterized by necrosis in margins of young developing leaves. It has been extensively studied that the factors including cultivars, low air flow in a controlled environment, small temperature difference, RH and other factors could cause tip burn, however, it is difficult to prevent tip burn occurrence in advance (Ki, Kee & Yong, 2000; Omasa, Nouchi & Kok, 2005; Lee et al., 2013; Lee et al., 2019; Maruo & Johkan, 2020; Birlanga, Acosta-Motos & Pérez-Pérez, 2021; Carotti et al., 2021). The RH is one of the widely accepted factors affecting tip burn occurrence. Ki & Yong-Beom (2008) reported that at 80%/80% (day/night) RH, the incidence of tip burn in butterhead-type lettuce ‘Omega’ is 100%; but at 40%/80% (day/night) RH, the incidence is zero; however, tip burn was not observed in leaf-type lettuce ‘Grandrapid’ at varied RH. Studies of Lisianthus also have shown that the occurrence of tip burn is not always associated with high humidity. Among five Lisianthus cultivars, the two cultivars grown at 70% RH showed significantly higher tip burn severity than the plants at 50% RH, but the tip burn severity of the other three cultivars was not alleviated by RH (Kuronuma et al., 2018).

In this study, a total of 30 vegetable varieties, representing eight families and 25 species, were grown in a closed plant factory to investigate plant adaptive performance. Unpredictably, 20 vegetable crops from 14 species and four families, exhibited different degrees of leaf damage, especially in cruciferous vegetables. Necrosis in margin of lower leaves occurred first, then the entire lower leaves gradually browned. The burning developed to the upper leaves gradually, until the entire plant’s leaves withered and died. This new physiological disorder is defined as “leaf burn” and that it has significant differences contrasted with tip burn. Tip burn is described as “leaf apex necrosis or water-soaking in young growing inner leaves”. To address this new question, we chose the flowering Chinese cabbage, a typical cruciferous vegetable in South China, as the research object. We hypothesize that different light conditions and relative air humidity suppress leaf burn incidence. Leaf burn incidence was the primary indicator to evaluate the alleviation effects. On this basis, leaf water potential, leaf temperature, stomatal behavior, and photosynthesis were furthermore investigated. We expect that the results will provide mechanistic insights into the causes of leaf burnt and optimize the light and relative humidity conditions for high-quality leafy vegetables production in the plant factory.

## Materials & Methods

### Plant material and growth conditions

All experiments in this study, from seed treatment to vegetable crop growth, were carried out in a small, closed plant factory with artificial light in the College of Horticulture, South China Agricultural University. The seedling module and the culture module (Sananbio China, Xiamen, Fujian, China), were installed in the plant factory; both of them are multi-layer vertical deep flow hydroponic systems. The main parameters of modules and light sources are shown in Table S1 and Fig. S1. The temperature, ventilation and the RH in the plant factory were controlled by an air conditioner system (KFR-120TW/(1256S) NhBa-2, Gree, China) and dehumidifiers (DY-620EB, Deye, Ningbo, Zhejiang, China). A constant temperature of 24 °C was set to ensure that the plant canopy temperature was stayed around at 26 °C when the lights were turned on. RH was set to 70  ± 10%. The canopy air velocity in plant factory was almost zero.

The seeds were imbibed in 55 °C warm water for 15 min, then soaked for 2 h with water, and germinated for the radicle to emerge under dark conditions. The radicle-emerging seeds were sowed into the seedling tray (60 cm × 29.5 cm × 4.9 cm) containing a sponge block with water, and then placed on the seedling module for 14 days. Once the cotyledons were flattened, the water was replaced with 1-strength Hoagland’s nutrient solution every three days in the nursery tray.

### Cultivation tests of vegetable species and cultivars in plant factory

Thirty types of common vegetable crops, from 25 species and eight families, were selected for this part. The 14-day-old seedlings grown on the seedling module were transplanted to the culture module for further hydroponics. The photoperiod was set to 12 h/12 h (day/night). The sampling time was determined according to the growth rate of different crops and the incidence of “leaf burn”, and photographs were taken accordingly.

Based on the degree of leaf burn of the different vegetable species, the degree of leaf burn was categorized into three grades: LV1, the plant does not show leaf burn during the whole growth period, and its growth and development are normal; LV2, the plant has a slight leaf burn, and the rate of new healthy leaf production is higher than that of the leaf burn of the lower leaves, *i.e.,* the number of healthy leaves is always more than burnt leaves, and the plants could grow continuously until harvest. LV3, severe leaf burn occurs in the plant, and the leaf burn rate of the lower leaves is higher than the new leaf production rate, the plant does not grow properly and eventually die.

### Light intensity, and photoperiod treatment

In order to analyze whether leaf burn occurs due to light conditions, two flowering Chinese cabbage varieties, Sijiu-19 (Sijiu, early-maturity cultivar) and Chixin No. 2 (Chixin, late-maturity cultivar), were examined to investigate the leaf burn incidence at various light intensities and photoperiod conditions in plant factory. The seedlings were planted in seedling trays, and then placed on the seedling module for 14 days. Shading nets were used to adjust the light intensity, and tin foil was used as seedling tray cover to adjust the photoperiod. The light treatments are shown in Table 1.

**Table 1:** Light intensities and photoperiod treatments.

Treatments	Typical PPFD at plant sites (µmol m^−2^ s^−1^)	Photoperiod (h d^−1^)	Daily light integral (mol m^−2^ d^−1^)
T1	150	12	6.48
T2	105	12	4.54
T3	105	10	3.78
T4	105	8	3.02

**Notes.**

PPFD Photosynthetic Photon Flux Density

### Relative air humidity treatment

Considering insignificant improvement of light conditions on the leaf burn occurrence of two flowering Chinese cabbage cultivars, we extended the analysis of environment factor RH on the prevention of leaf burn occurrence. The 70 ± 10% of canopy RH was set as the control treatment. The sealed seedling tray cover was used to increase the canopy RH to 90–99% (daily average VPD 0.16 kPa). The control seedlings were covered with seedling tray lid with only the top and four corners. All the seedlings were grown in seedling trays, and then placed in the seedling module with a photoperiod of 12 h/12 h L to D and a constant temperature of 24 °C within 16 days. Seventeen days after sowing, the seedlings were transplanted to culture module with hydroponic system for continuous growth until the harvest of early-maturity cultivar ‘Sijiu’ with a photoperiod of 12 h/12 h L to D and a constant temperature of 24 °C. Unfortunately, the seedlings gradually died grown at 70% RH; at 90% RH, both two cultivars grew healthy. Chlorophyll content and leaf temperature were examined on the 14th day after sowing. Leaf water potential, stomatal density, stomatal aperture, and photosynthetic gas exchange parameters were studied on the 12th, 14th, and 16th days after sowing. Growth-related parameters were determinated on the 14th and 35th days, and quality indicators were measured on 35th days.

### Statistics of leaf burn incidence and measurement of growth parameters

The leaf burn incidence is defined as the ratio of the number of plants with leaf burn to total number of plants (leaf burn incidence = numbers of plants with burnt leaves in any extent/total number of plants). The fresh weight of the whole plant was weighed with electronic balance. The plant dry weight was obtained after drying the fresh plants in an oven at 100 °C for 30 min, followed by drying at 80 °C to constant weight.

### Determination of chlorophyll pigment content

The chlorophyll pigment contents were examined referring to the method of Wang et al. (2022). Briefly, 0.2 g fresh leaf samples were soaked in 20 mL of an acetone-ethanol mixture (acetone: ethanol = 2:1, v/v) for 24 h at 25 °C in the dark till the leaves turned white. The absorbance of the supernatant was determined by UV-spectrophotometer (Shimadzu UV-16A, Shimadzu, Corporation, Kyoto, Japan) at 645 nm, 663 nm, and 440 nm, respectively.

### Determination of quality index

The quality index, including vitamin C (Vc), soluble sugar and soluble protein, was determined in the flower stalk of cultivar ‘Sijiu’, and in the rosette leaves of cultivar ‘Chixin’. The leaves were separated from the flower stalk, and the two parts were used for analysis respectively in cultivar ‘Sijiu’.

Vc content was determined by molybdenum blue colorimetric method (Tan et al., 2021). Briefly, fresh leaf and flower stalk samples (1.0 g) were extracted using 25 mL of oxalic acid EDTA solution, and then one mL of supernatant was mixed with 0.5 mL of phosphate-acetic acid, two mL of 5% ammonium molybdate. After incubation at 30  °C for 15 min, the absorbance was recorded at 760 nm using a UV-spectrophotometer (Shimadzu UV-16A, Shimadzu, Corporation, Kyoto, Japan).

Soluble sugar content was determined using anthrone-sulfuric acid colorimetric method (Kohyama & Nishinari, 1991). Fresh leaf and flower stalk samples were heated in a boiling water bath with 25 mL of distilled water for 30 min. Then, 0.5 mL of supernatant was mixed with 1.5 mL distilled water, 0.5 mL anthrone ethyl acetate, and five mL vitriol. The absorbance was recorded at 630 nm using a UV-spectrophotometer (Shimadzu UV-16A, Shimadzu, Corporation, Kyoto, Japan).

Soluble protein content was measured by Coomassie brilliant blue G-250 dye method (Bradford, 1976). Fresh leaf and flower stalk samples (1.0 g) were homogenized with eight mL distilled water. The homogenate was centrifuged at 8,000 ×*g* for 10 min at 4 °C; then, 0.2 mL the supernatant was mixed with 0.8 mL distilled water and five mL 0.1 g/L Coomassie brilliant blue G-250 solution. The absorbance was measured at 595 nm using a UV-spectrophotometer (Shimadzu UV-16A, Shimadzu, Corporation, Kyoto, Japan) after standing for 15 min.

### Determination of leaf water potential

Leaf water potential was examined using a WP4C dewpoint potential meter (Original Decagon, Pullman, Washington, USA) (Lu et al., 2021). Briefly, the leaves were sampled and quickly placed into the sample chamber to make sure the leaves to cover the full bottom of the sample chamber. The difference between the sample temperature and the block chamber temperature was kept below −0.5 °C.

### Observation of stomatal aperture and density

The scotch tape method was used to obtain the leaf lower epidermis. Stomata image from an epifluorescence microscope (Olympus BX53, Olympus Corporation, Tokyo, Japan) was analyzed by ImageJ software (Rasband, W.S., ImageJ 1.8.0 172, U.S.A) to count the number of stomata and measure the aperture size (Lawrence et al., 2018).

### Determination of photosynthetic parameters

The seedlings were adapted about 0.5 h under darkness to estimate the maximum quantum yield of photosystem II photochemistry (Fv/Fm) using an Imaging-PAM chlorophyll fluorometer (PlantExplorer, PhenoVation, Wageningen, Netherlands). Gas exchange measurements were performed using a portable photosynthesis system (LI-6400, LI-COR, USA). The net photosynthetic rate, intercellular CO_2_ concentration, stomatal conductance, and transpiration rate of the first true leaf were measured after turning on the light for 6 h in the morning. photosynthetic photon flux density (PPFD) set as 200 µmol m^−2^ s^−1^. The portable leaf area meter (RX-YMJ, TuoPu, Zhejiang, China) was used to determine the actual blade area enclosed in the leaf chamber, and the gas exchange area were recalculated (Carriquí, Nadal & Flexas, 2021).

### Data analysis

Leaf burn incidence and growth determinations were performed with 45 and 15 replicates per treatment, respectively. Leaf water potential, leaf temperature and quality index were carried out with five replicates. Leaf stomatal parameters were performed with six replicates. Fv/Fm was done with 18 replicates. Photosynthetic parameters were performed with three replicates. All experiment was conducted based on a randomized complete block design with biological repeats. The data are presented as the mean ± SEs and were analyzed by SPSS 20 statistics software (IBM Corp., Armonk, NY, USA), the experimental data were analyzed with Duncan’s multiple range test at a significance level of 0.05 and 0.01.

## Results

### Differences in the degree of leaf burn among vegetable species and varieties under the closed plant factory

Among the 30 vegetable varieties studied, 10 varieties performed normal growth and development, including Cucurbitaceae family plants, *Lagenaria siceraria* Stand var. siceraria, *Benincasa hispida* Cogn. var. chieh-qua How, and *Luffa acutangular* Roxb; the Asteraceae family plants, *Chrysanthemum coronarium* L. var. spatiosum Bailey and *Lactuca sativa* L. var. longifolia Lam; a Solanaceae family plant, *Solanum lycopersicum* L.; an Amaranthaceae family plant, *Amaranthus mangostanus* L.; a Chenopodiaceae family plant, *Beta vulgaris* L. var. cicla Koch.; an Lamiaceae family plant, *Ocimum basilum* L. No leaf burn (LV1) was observed in these vegetables.

**Figure 1:**
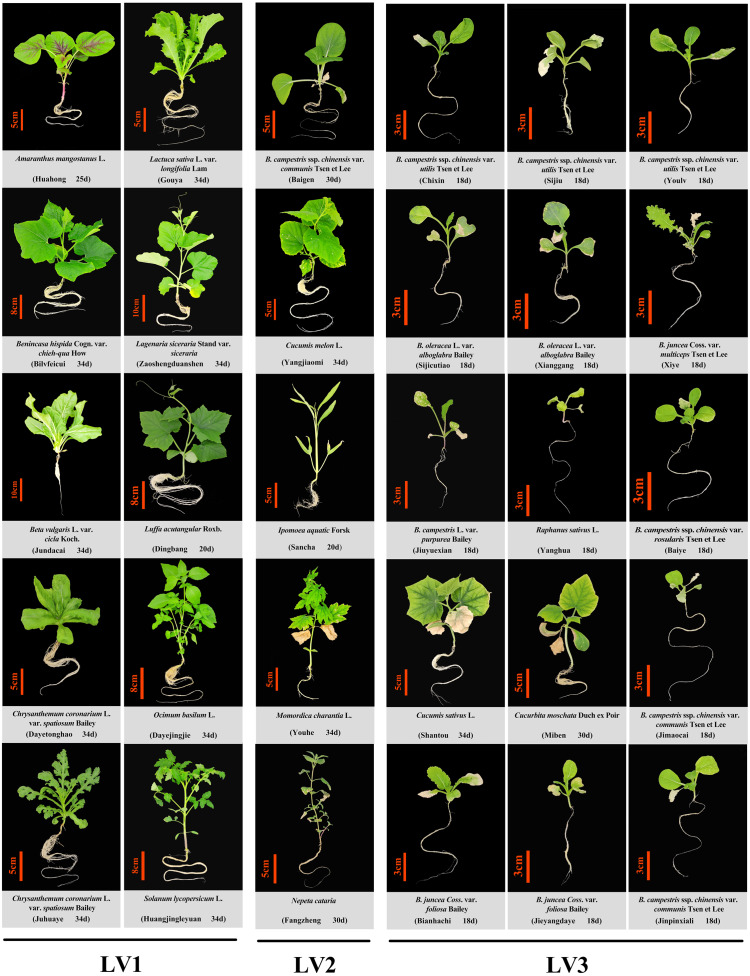
Phenotype of 30 common vegetable crops cultured in a closed plant factory under artificial lighting. D, days after sowing; *B. campestris*, *Brassica campestris*; *B. oleracea*, *Brassica oleracea*; *B. juncea*, *Brassica juncea*. LV1, the plant does not show leaf burn during the whole growth period, and its growth and development are normal; LV2, the plant has a slight leaf burn, and the rate of new healthy leaf production is higher than that of the leaf burn of the lower leaves, *i.e.,* the number of healthy leaves is always more than burnt leaves, and the plants could grow continuously until harvest. LV3, severe leaf burn occurs in the plant, and the leaf burn rate of the lower leaves is higher than the new leaf production rate, the plant does not grow properly and eventually die.

The remaining 20 vegetable varieties all showed different degrees of leaf burn. Occurrence of leaf burn in the cruciferous vegetables was the most common and severe, except *Brassica campestris* ssp. chinensis var. communis Tsen et Lee (LV2 degree), all other cruciferous vegetables showed severe leaf margin browning and death (LV3), and could not grow and develop normally, and eventually died. Cucurbitaceae species showed obviously different leaf burn resistance. *Momordica charantia* L., and *Cucumis melon* L. exhibited minor leaf burn symptoms (LV2), while *Cucumis sativus* L. and *Cucurbita moschata* Duch ex Poir had severe leaf burn (LV3) (Table 2, Fig. 1).

**Table 2:** Severity levels of leaf burn of 30 vegetable cultivars grown in a closed plant factory.

Family	Species and variety	Cultivar	Sampling time (days after sowing)	Severity levels of leaf burn
Amaranthaceae	*Amaranthus mangostanus* L.	Huahong	25	LV1
Asteraceae	*Chrysanthemum coronarium* L. var. *spatiosum* Bailey	Dayetonghao	34		
			Juhuaye		34	
	*Lactuca sativa* L. var. *longifolia* Lam	Gouya	34		
Chenopodiaceae	*Beta vulgaris* L. var. *cicla* Koch.	Jundacai	34		
Cucurbitaceae	*Lagenaria siceraria* Stand var. *siceraria*	Zaoshengduanshen	34		
		*Benincasa hispida* Cogn. var. *chieh-qua* How	Bilvfeicui		34	
	*Luffa acutangular* Roxb.	Dingbang	20		
Lamiaceae	*Ocimum basilum* L.	Dayejingjie	34		
Solanaceae	*Solanum lycopersicum* L.	Huangjingleyuan	34	
Brassicaceae	*Brassica campestris* ssp. *chinensis* var. *communis* Tsen et Lee	Baigen	30	LV2
Convolvulaceae	*Ipomoea aquatic* Forsk	Sancha	20		
Cucurbitaceae	*Momordica charantia* L.	Youhe	34		
	*Cucumis melon* L.	Yangjiaomi	34		
Lamiaceae	*Nepeta cataria*	Fangzheng	30	
Brassicaceae	*Brassica campestris* ssp. *chinensis* var. *utilis* Tsen et Lee	Chixin	18	LV3
					Sijiu	18	
			Youlv		18	
		*Brassica oleracea* L. var. *alboglabra* Bailey	Sijicutiao		18	
			Xianggang		18	
		*Brassica juncea* Coss. var. *foliosa* Bailey	Bianhachi		18	
			Jieyangdaye		18	
		*Brassica campestris* ssp. *chinensis* var. *communis* Tsen et Lee	Jinpinxiali		18	
			Jimaocai		18	
		*Brassica juncea* Coss. var. *multiceps* Tsen et Lee	Xiye		18	
		*Brassica campestris* L. var. *purpurea* Bailey	Jiuyuexian		18	
		*Raphanus sativus* L.	Yanghua		18	
	*Brassica campestris* ssp. *chinensis* var. *rosularis* Tsen et Lee	Baiye	18		
Cucurbitaceae	*Cucumis sativus* L.	Shantou	34		
	*Cucurbita moschata* Duch ex Poir	Miben	30	

### The incidence of leaf burn in flowering Chinese cabbage under different light intensity and photoperiod conditions

**Figure 2:**
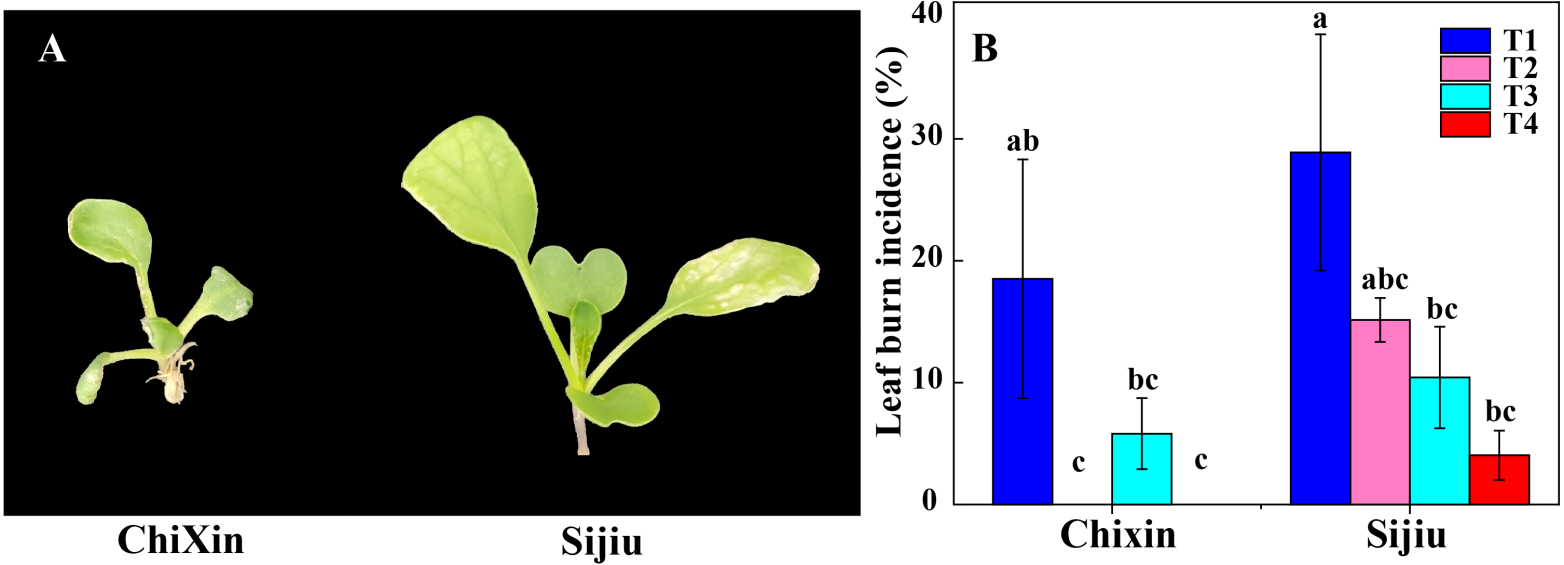
Leaf burn incidence of two flowering Chinese cabbage cultivars cultured in plant factory response to different light conditions. (A) Plant phenotype with varied degree of leaf burn under PPFD 150 µmol m^−2^ s^−1^ at 12 h d^−1^ conditions. (B) The incidence of leaf burn. T1, PPFD 150 µmol m^−2^ s^−1^ at 12 h d^−1^; T2, T3 and T4, PPFD 105 µmol m^−2^ s^−1^ at 12, 10, and 8 h d^−1^ respectively. Values represent the mean ± standard error (*n* = 45), different lowercase letters indicate significant differences among different treatments and cultivars at a significant level of 0.05 using Duncan’s multiple range tests.

Under different light conditions, cultivar ‘Chixin’ was more resistance to leaf burn compared with the cultivar ‘Sijiu’. Under PPFD 150 µmol m^−2^ s^−1^ and 12 h d^−1^ photoperiod (T1), two flowering Chinese cabbage cultivars exhibited different degrees of leaf burn symptom, and leaf burn incidence reached 28.89% and 18.52% in ‘Sijiu’ and ‘Chixin’, respectively (Fig. 2). Decreasing light intensity from PPFD 150 µmol m^−2^ s^−1^ (T1) to 105 µmol m^−2^ s^−1^ (T2) reduced the incidence to 15.21% in ‘Sijiu’, and absolutely inhibited leaf burn occurrence in ‘Chixin’ (Fig. 2B). When the photoperiod was shortened from 12 to 8 h d^−1^ at 105 µmol m^−2^s^−1^, leaf burn incidence gradually reduced in ‘Sijiu’. The cultivar ‘Chixin’ did not show any leaf burn symptom at 12 h d^−1^ (T2) and 8 h d^−1^ (T4) photoperiod and exhibited mild leaf damage at 10 h d^−1^ (T3), with 5.88% of leaf burn incidence. Although leaf burn occurrence was affected to different degrees under different light conditions, all plants grew slowly and bad in general.

### The effect of RH on the leaf burn occurrence and physiological characteristics of flowering Chinese cabbage

**Figure 3:**
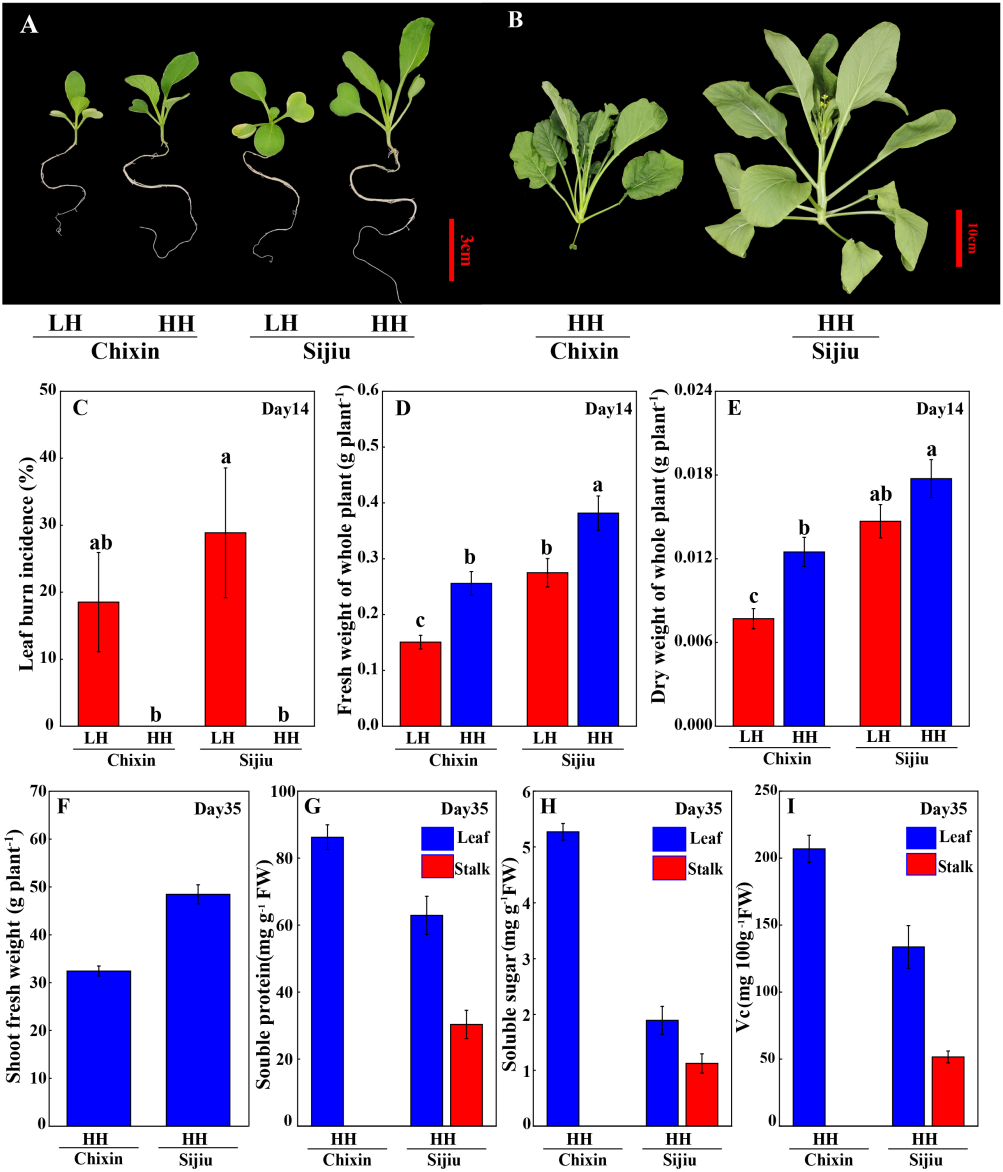
Leaf burn occurrence and plant growth of two flowering Chinese cabbage cultivars grown in plant factory response to different relative air humidity. (A) Plant phenotype under low and high relative air humidity conditions on 14 days after sowing. (B) Plant phenotype under high relative air humidity conditions on 35 days after sowing. (C) Leaf burn incidence on 14 days after sowing (*n* = 45). (D) Fresh weight of whole plant on 14 days after sowing (*n* = 15). (E) Dry weight of whole plant on 14 days after sowing (*n* = 15). (F) Shoot fresh weight of plants under high relative air humidity conditions on 35 days after sowing. (G) Soluble protein contents of plants under high relative air humidity conditions on 35 days after sowing. (H) Soluble sugar contents of plants under high relative air humidity conditions on 35 days after sowing. (I) Vitamin C contents of plants under high relative air humidity conditions on 35 days after sowing. LH, low relative air humidity; HH, high relative air humidity. Values are means ± standard error (*n*, sample replicates), different letters indicate significant differences among different treatments and cultivars at a significance level of 0.05 using Duncan’s multiple range tests.

Because the minor alleviation of light conditions on the leaf burn occurrence and plant growth in flowering Chinese cabbage, we attempted to explore the effect of environment factor RH. Fortunately, both in ‘Sijiu’ and ‘Chixin’, increasing RH from 70% to 90% by putting a cover on the seedling tray not only absolutely prevented the occurrence of leaf burn (Figs. 3A∼3C), but also improved plant growth; as a result, plant fresh weight and dry weight were greatly promoted. Fourteen days after sowing, fresh weight of plants cultivated at high RH level increased by 38.79% and 69.76%, and dry weight of plants increased by 20.76% and 62.21% respectively in ‘Sijiu’ and ‘Chixin’ compared with the plants cultivated at low RH (Figs. 3D∼3E). Fifteen days after sowing, ‘Chixin’ and ‘Sijiu’ were transplanted to the culture module for continuous growth. At high RH level, two flowering Chinese cabbage cultivars grew healthy and developed normally without leaf burn symptom (Fig. 3B); thirty-five days after sowing, the early-maturing cultivar ‘Sijiu’ formed flower stalks, and the late-maturing cultivar ‘Chixin’ was still at seedling stage. In ‘Sijiu’, shoot fresh weight was 48.53 g plant^−1^, and the contents of soluble sugar, Vc and soluble protein were 1.89 mg gFW^−1^, 133.71 mg 100 gFW^−1^ and 62.89 mg gFW^−1^ respectively in the leaves, and 1.12 mg gFW^−1^, 51.63 mg 100gFW^−1^ and 30.35 mg gFW^−1^ respectively in the stalks on days 35 after sowing under high RH conditions; In ‘Chixin’, shoot fresh weight was 32.43 g plant^−1^, and the contents of soluble sugar, Vc and soluble protein were 5.27 mg gFW^−1^, 206.91 mg 100gFW^−1^ and 86.24 mg gFW^−1^ respectively in the leaves (Figs. 3G∼3I). At low RH, both the cultivar ‘Sijiu’ and ‘Chixin’ gradually died after transplantation to the culture module.

**Figure 4:**
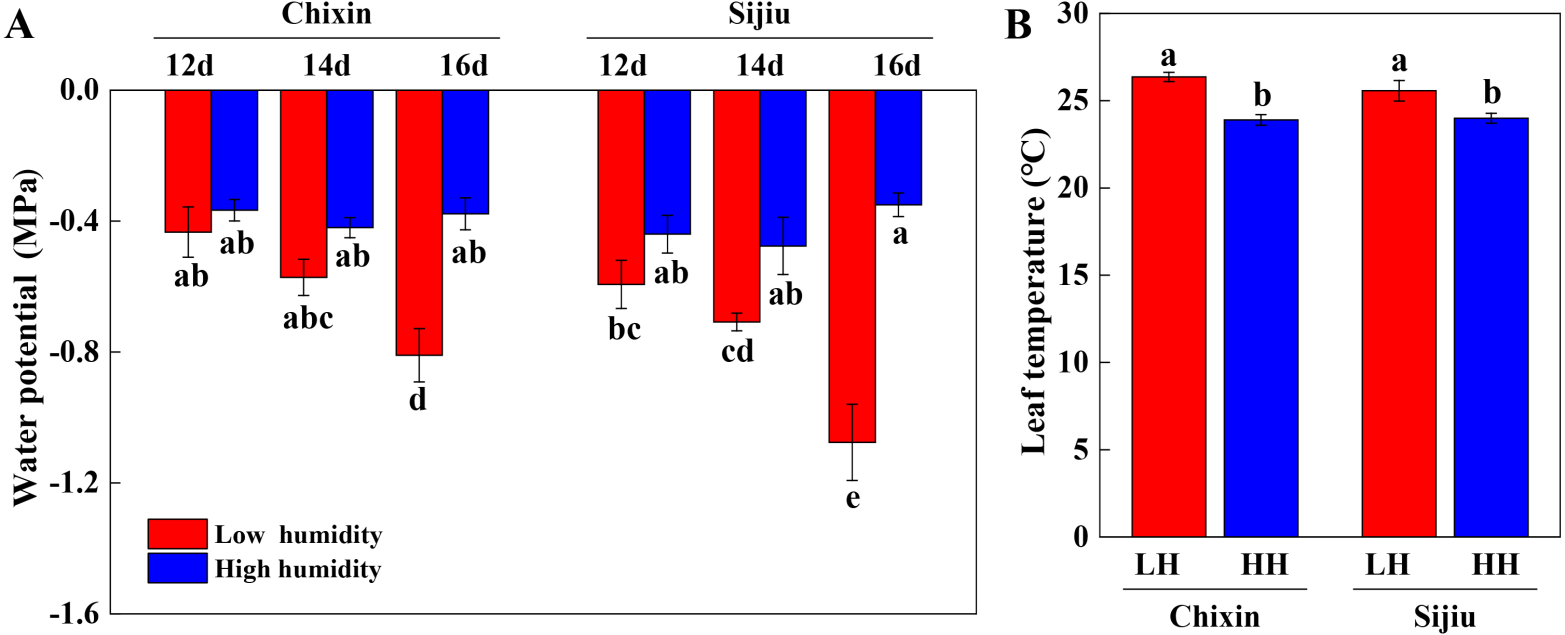
Leaf water potential (A) on 12, 14 and 16 days after sowing and leaf temperature (B) on 14 days after sowing in two flowering Chinese cabbage cultivars grown in plant factory response to different relative air humidity respectively. LH, low relative air humidity; HH, high relative air humidity. Values represent the mean ± standard error (*n* = 5), different letters indicate significant differences among different treatments and cultivars at a significance level of 0.05 using Duncan’s multiple range tests.

We extended the analysis of leaf water potential using a WP4C dewpoint potential meter. At low RH, the water potential in the leaves of two cultivars gradually decreased with the extension of the experiment, which decreased faster in ‘Sijiu’ than ‘Chixin’. On day 16 after sowing, the leaf water potential in ‘Sijiu’ was significantly lower than that in ‘Chixin’. Conversely, the leaf water potential both in ‘Sijiu’ and ‘Chixin’ kept relatively stable under high RH conditions. Compared with the plants grown at low RH level, the significantly higher water potential was maintained in the leaves of ‘Sijiu’ grown at high RH level on 16 days after sowing, and in the leaves of ‘Chixin’ on days 14 and 16 after sowing (Fig. 4A). On day 14 after sowing, the significantly lower T_leaf_ was observed in the plants at high RH than the plants at low RH regardless of plant varieties (Fig. 4B).

**Figure 5:**
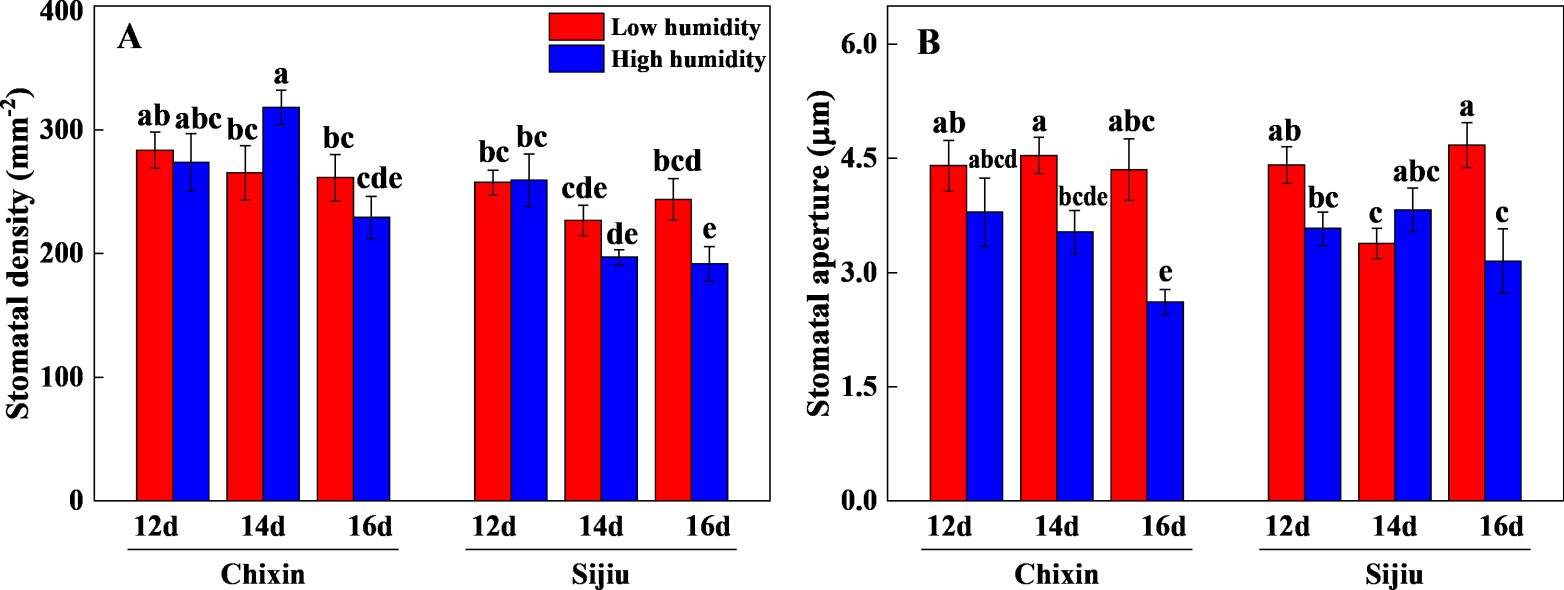
Leaf stomatal density (A) and aperture (B) of two flowering Chinese cabbage cultivars grown in a closed plant factory response to different relative air humidity on 12, 14 and 16 days after sowing. LH, low relative air humidity; HH, high relative air humidity. Values represent the mean ± SE (*n* = 6), different lowercase letters indicate significant differences among different treatments and cultivars at a significance level of 0.05 using Duncan’s multiple range tests.

The stomatal aperture and density of plants were generally reduced in response to the increased RH. On 16 days after sowing, high RH significantly reduced the stomatal aperture and density in ‘Sijiu’ leaves and stomatal aperture in ‘Chixin’ leaves. Except that on day 14 after sowing, high RH slightly increased the stomatal density in ‘Chixin’ leaves and stomatal aperture in ‘Sijiu’ leaves compared to low RH treatment (Fig. 5).

**Figure 6:**
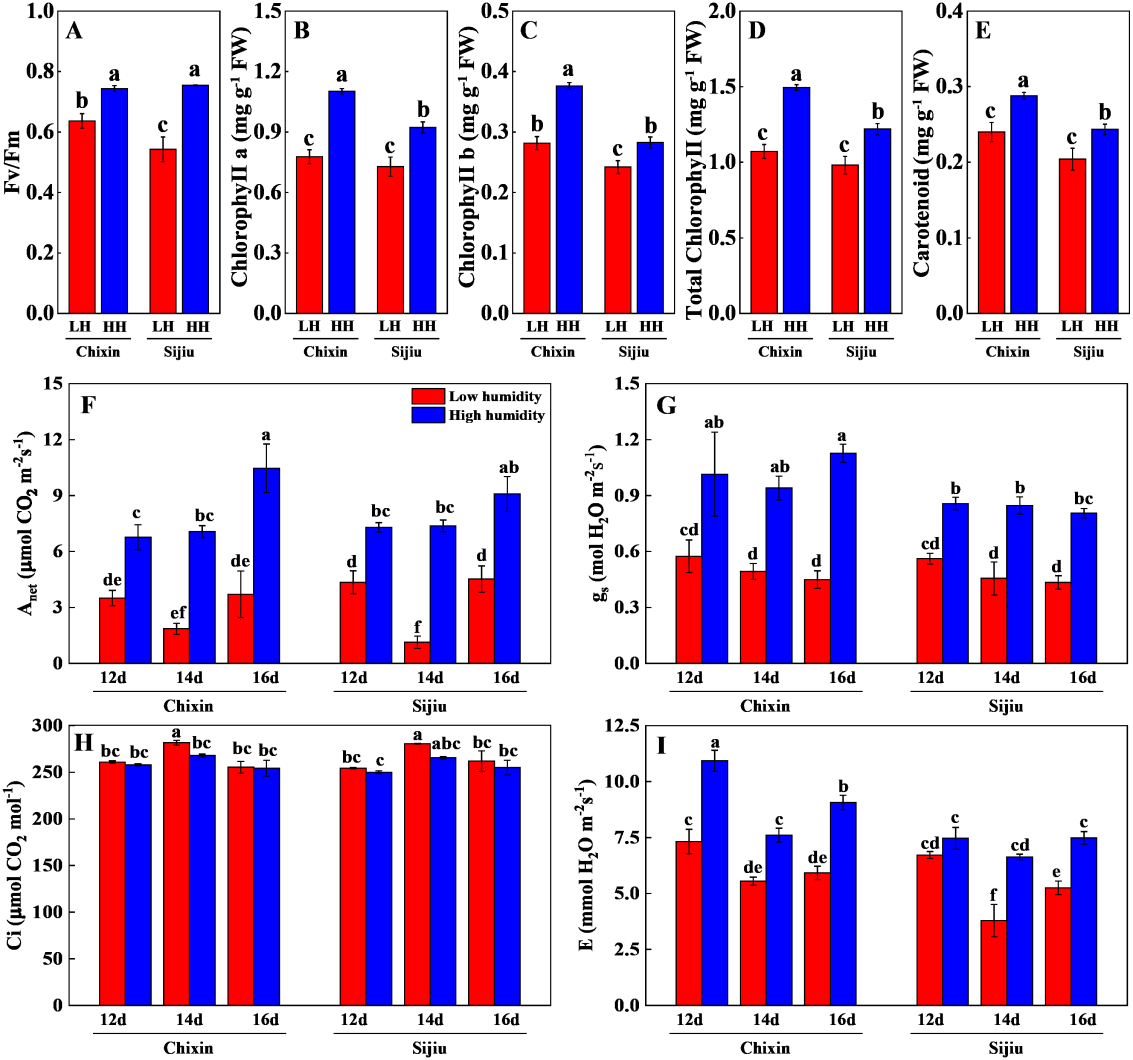
Changes of photosynthetic parameters in two flowering Chinese cabbage cultivars grown in plant factory response to different relative air humidity. (A) Fv/Fm (*n* = 18). (B–E) Photosynthetic pigment contents (*n* = 3). (F) Anet (*n* = 3). (G) Gs (*n* = 3). (H) Ci, (*n* = 3). (I) Transpiration (*n* = 3). Values represent the mean ± standard error (*n*, sample replicates), different lowercase letters indicate significant differences among different treatments and cultivars at a significance level of 0.05 using Duncan’s multiple range tests.

The photosynthetic performance of flowering Chinese cabbage grown at 90% RH was significantly improved compared with those at 70% RH. The high RH increased maximum quantum yield of PS II (Fv/ Fm), the levels of chlorophyll a, chlorophyll b, carotenoids, and total chlorophyll in comparison to low RH treatment on day 14 after sowing (Figs. 6A∼6E). Compared with the plants cultivated at low RH, the stomatal conductance in ‘Chixin’ leaves cultivated at high RH increased by 76.77%, 90.74%, and 150.74% respectively, and increased by 52.58%, 85.95% and 85.63% respectively in ‘Sijiu’ leaves 12, 14 and 16 days after sowing; the transpiration rate in ‘Chixin’ leaves treated with high RH increased by 49.29%, 37.06% and 53.15%, and increased by 11.17%, 75.17% and 42.54% in ‘Sijiu’ leaves respectively; the net photosynthetic rate (Pn) in ‘Chixin’ leaves increased by 93.08%, 280.59%, and 182.52%, and increased by 67.84%, 553.71% and 101.00% in ‘Sijiu’ leaves respectively. Under two RH levels, no significant difference of internal CO_2_ (C_i_) was observed within the same cultivar at the same sample time; C_i_ was maintained at about 250 µmol CO_2_.mol^−1^ (Figs. 6F∼6I). In general, no great difference of the photosynthetic performance was observed between two cultivars regardless of canopy RH, although higher Fv/Fm, chlorophyll b and transpiration rate in ‘Chixin’ leaves at low RH were found than those in ‘Sijiu’.

## Discussion

In recent years, the market demand for locally grown agricultural products has gradually increased; therefore, research on the technologies related to controlled environment agriculture has attracted much more attention. The introduction of commercial light-emitting diodes (LEDs) as plant light sources is one of the major factors driving the application of CEA to urban food production. The properties of LED, *i.e.,* narrow-spectrum emissions, small size, and cool photon-emitting surfaces, permit close canopy lighting without leaf burn. The indoor plant factory using LEDs as the only light source is an important form of CEA, which is widely used in the production of leafy green vegetables represented by lettuce, as well as ornamental plants, and so on.

In this study, under the LEDs condition in plant factory, 20 out of 30 kinds of vegetable cultivars appeared leaf burn symptom to varied degree, specifically, yellowing at the leaf margin of the old leaves firstly, then the whole leaves of the old leaves, and then the upper leaves (Table 2, Fig. 1), eventually the whole plant leaves withered and died. The leaf burn phenomenon was nearly studied until now, possibly because of only a few kinds of vegetable species widely produced in large scale in the plant factory. The leaf burn symptom was different from the tip burn phenomenon extensively studied in lettuce. Tip burn is a physiological disorder involving necrosis or water-soaking at the leaf apex of young developing inner leaves, which mainly due to calcium deficiency. It was also found that tip burn occurrence was genotype-dependent, and common in lettuce, strawberry, cabbage and Chinese cabbage in plant factory (Saure, 1998; Maruo & Johkan, 2020). The occurrence of leaf burn was also genotype-dependent, and the most common and severe in cruciferous leafy vegetables, especially flowering Chinese cabbage and radish (Table 2, Fig. 1).

Extensive research has been conducted on the influence of LED light intensity, spectrum, and photoperiods in horticultural crop yield, nutritional quality, and pest and/or disease control, especially in the production of green leafy vegetables (Bian, Yang & Liu, 2015; Mitchell et al., 2015; Gómez et al., 2019). Sago (2016) reported that a positive relationship between light intensity and the tip burn occurrence in butterhead lettuce at PPFD 150, 200, 250, and 300 µmol m^−2^ s^−1^. Lee et al. (2019) found that the influence of light intensity on the tip burn incidence was dependent on the cultivar type in crisphead lettuce. At PPFD 150, 200, and 250 µmol m^−2^ s^−1^, the incidence of tip burn in cultivar ‘Adam’ increased linearly with the increase of light intensity in the low-temperature condition, whereas the incidence was the lowest in cultivar ‘Sensation’ at the highest light intensities. In cultivar ‘Manchu’, there was non-significant association between tip burn incidence and light intensity. LED light conditions, that may have roles in mitigating leaf burn in green leafy vegetables, but have not been identified, and this assumption needs to be further confirmed. Therefore, two typical flowering Chinese cabbage cultivars, one early maturing and another late maturing variety, were selected to further examine the effects of light intensities and photoperiods with daily light integral (DLI) from 3.02 to 6.48 mol m^−2^ d^−1^ on leaf burn occurrence. Unexpectedly, changing light intensities and photoperiod in the closed plant factory only had minor effect on suppression of leaf burn occurrence. The leaf burn occurrence in ‘Sijiu’ decreased linearly with the decrease of light intensity and shortening of photoperiod, however, in ‘Chixin’, no association was observed between leaf burn incidence and photoperiod (Fig. 2). These results argued against an important role of light-influenced tip burn phenomenon (Sago, 2016; Lee et al., 2019).

Water is not only an important resource necessary for plant survival, growth, and distribution, but also is a critical mediator for carbon assimilation, signal transduction, photosynthetic transport, and distribution. The kinetics of plant-water transport reflect plant health and response to environmental fluctuations situation (Zhou et al., 2021). Environment air humidity is recognized as affecting the development of leaf tip burn disorder. The prevailing view is that high humidity favors the tip burn occurrence (Islam et al., 2004; Ki & Yong-Beom, 2008; Kuronuma et al., 2018), although there are a few contradictory findings in some species or cultivars (San Bautista et al., 2020). On the contrary, our results showed that an increasing of RH can completely relieve the phenotype of leaf burn in the plant factory regardless of genotype. The shoot fresh weight and nutrient quality in early-maturity cultivar ‘Sijiu’ at high RH level reached the marketable weight and quality standard of hydroponic grown plants in the field, and the late-maturity cultivar ‘Chixin’ grew healthy during the whole experiment (Fig. 3) (Wang et al., 2022).

Leaf water potential is widely used as an important indicator of plant water status. A quadratic relationship is observed between the photosynthetic rate and leaf water potential, and the photosynthetic rate decreases in maize plants when leaf water potential reduced from 84.11% to 68.26% (Zhou et al., 2021). Optimal adjustment of water loss through fine-tuning of stomatal behavior is elemental for plant survival especially upon increased evapotranspiration demand (Fanourakis et al., 2020). In controlled environments, many measurements are adopted to regulate air humidity to prevent leaf water loss, such as the application of a pad-fan systems, roof sprinklers, and fogging systems. Among them, the fogging system is considered to be an effective mean to alleviate the adverse effects of high-temperature on cherry tomato grown in greenhouses during summer, and thus increase the yield (Leyva et al., 2013). The increase of VPD in greenhouses in winter caused by ventilation at noontime reduced plant biomass and yield. Increasing air humidity by the application of fogging greatly lowered VPD from 1.4 kPa to 0.8 kPa in the midday, and consequently increased the stomatal density, stomatal index, gs, and net photosynthetic rate of tomato leaves, which led to an increase in the biomass and yield (Lu et al., 2015). In this study, increasing canopy RH from 70% to 90% decreased leaf stomatal aperture and density, thus hindered diffusional water loss, which helped to maintain leaf water potential together with high environmental RH. Conversely, under low RH conditions, the weakened stomatal closing response, indicated by higher leaf stomatal aperture and density, resulted in leaf water loss continuously, consequently caused leaf burnt (Figs. 4∼5). The higher water potential in leaves ensured that the leaves functioned properly (Damm et al., 2018), subsequently, plant growth parameters, *i.e.,* plant biomass, photosynthetic pigment, transpiration rate and Pn, were significantly improved (Fig. 6). Previous studies also confirmed the essential function of the leaf structure and stomatal behavior in photosynthetic acclimation to humidity, *i.e.,* the number of small stomata, epidermal and mesophyll cell size and stomatal and minor vein density (Du et al., 2019; Amitrano et al., 2021). In the sunny or overcast of natural environment without abiotic stress, a drop of transpiration rate was observed in the plants at high RH compared to the moderate RH, companied by increased T_leaf_ because of weakened evaporative cooling (Fanourakis et al., 2020). Our result was somewhat different from the results of Fanourakis et al. (2020), the higher transpiration rate was found under high RH conditions compared to low RH conditions. Nevertheless, the decreasing trend of transpiration rate was consistent at both RH levels with the extension of the experiment (Fig. 6). One possible explanation was that low RH in the plant factory caused the decline of transpiration rate and photosystem damage was similar to abiotic stress. In our previous studies, the transpiration rate was sharply reduced under high temperature, drought and other abiotic stress (Zhong et al., 2019; Jahan et al., 2021). Moreover, the increased environmental humidity in the air and higher transpiration rate of the plants at high RH level reduced plant canopy temperature in comparison to low RH treatment (Figs. 4 and 6I), as a result, attenuated the possible adverse effects of the excessive heat emitted by LED lights on the plants.

## Conclusions

**Figure 7:**
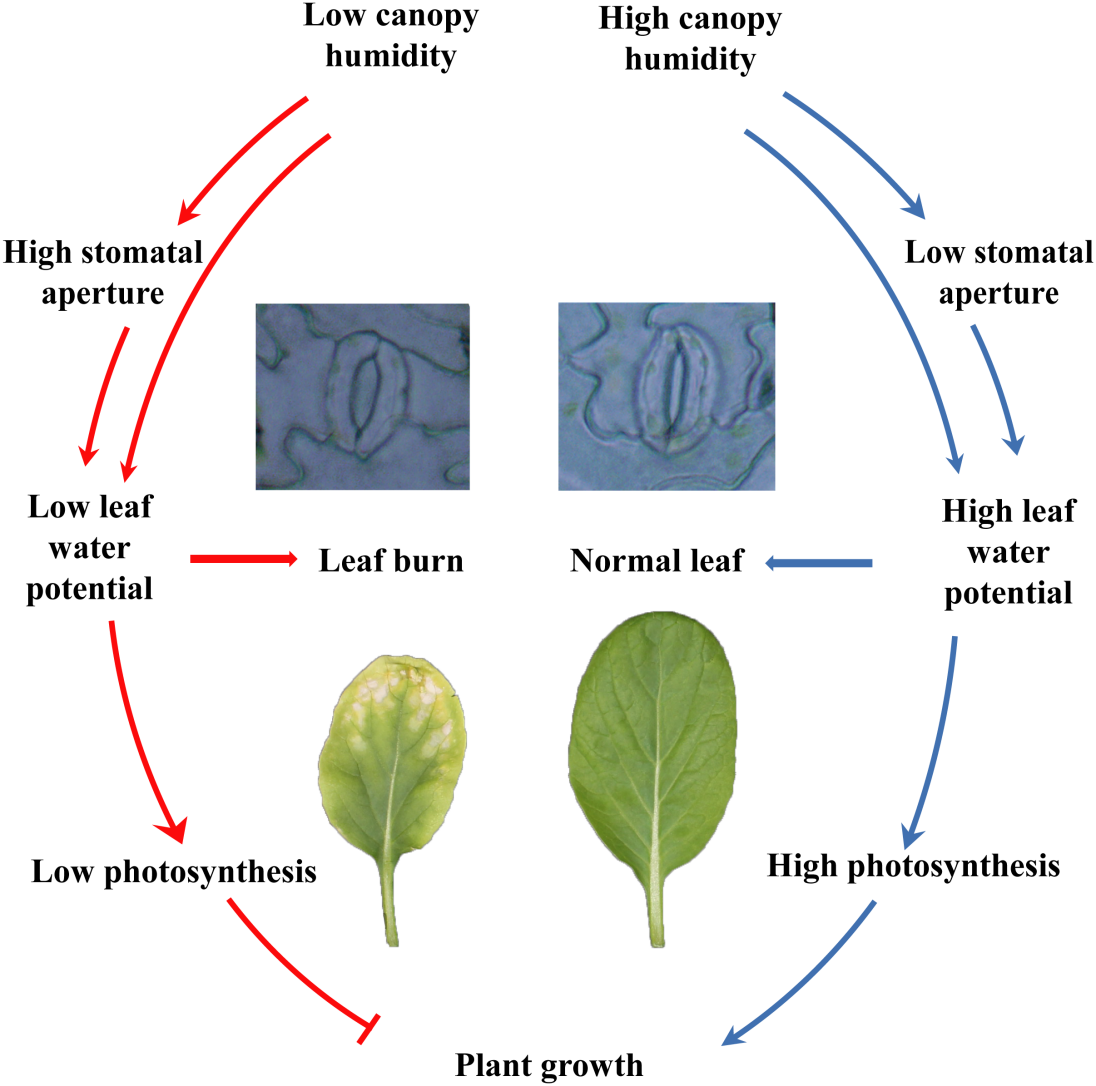
Model for the control of leaf burn occurrence by high relative air humidity.

In summary, the current study showed that different species and cultivars of the plants performed differently in closed plant factories, and cruciferous leafy vegetables had poor adaptability. Cruciferous leaf crops had severe leaf burn, which was reflected in plant growth, photosynthesis, etc. Furthermore, plants grew healthy at high canopy humidity, by maintaining lower stomatal aperture, higher water potential and leaf function and photosynthesis (Fig. 7). Although the relative air humidity in canopy have been shown to be a promising cure for keeping healthy growth of flowering Chinese cabbage, further study is needed to reveal the fine mechanisms between humidity and leaf burn.

##  Supplemental Information

10.7717/peerj.14325/supp-1 Table S1 Major parameters of the Radix module

10.7717/peerj.14325/supp-2 Figure S1 Spectral composition of the light-emitting diode (LED) in seedling module (A) and culture module (B)

10.7717/peerj.14325/supp-3 Data S1 Raw data

## Additional Information and Declarations

### Data Availability

The following information was supplied regarding data availability: The raw measurements are available in the Supplemental Files.
