# Supplementary material for: High relative humidity improves leaf burn resistance in flowering Chinese cabbage seedlings cultured in a closed plant factory
Source: PeerJ. 2022 Nov 8;10:e14325. doi: 10.7717/peerj.14325 (PMC9651049; doi:10.7717/peerj.14325)
Supplement: Table S1 [file peerj-10-14325-s001.docx]

|  | Seedling Module | Culture Module |
| --- | --- | --- |
| Equipment size (mm) | 1346*942*2706 | 1346*942*2863 |
| Layers | 7 | 6 |
| Light source | Sananbio “Flamingo” series  (model: ZK-TB18-VE02/A) | Sananbio “Flamingo” series  (model: ZK-TB15-GE02/A) |
| Light spectrum | 25% blue (400-500 nm),  55% Red (600-700 nm) | 15% blue (400-500nm), 60% Red (600-700 nm) |
| Light-to-Bed Height (mm) | 200 | 280 |
| Typical PPFD at plant sites (μmol·m^-2^·s^-1^) | 150 | 200 |
| Numbers of LED lights per layer | 6 | 6 |

PPFD, Photosynthetic Photon Flux Density
