# Supplementary figures and images for: High relative humidity improves leaf burn resistance in flowering Chinese cabbage seedlings cultured in a closed plant factory
Source: PeerJ. 2022 Nov 8;10:e14325. doi: 10.7717/peerj.14325 (PMC9651049; doi:10.7717/peerj.14325)

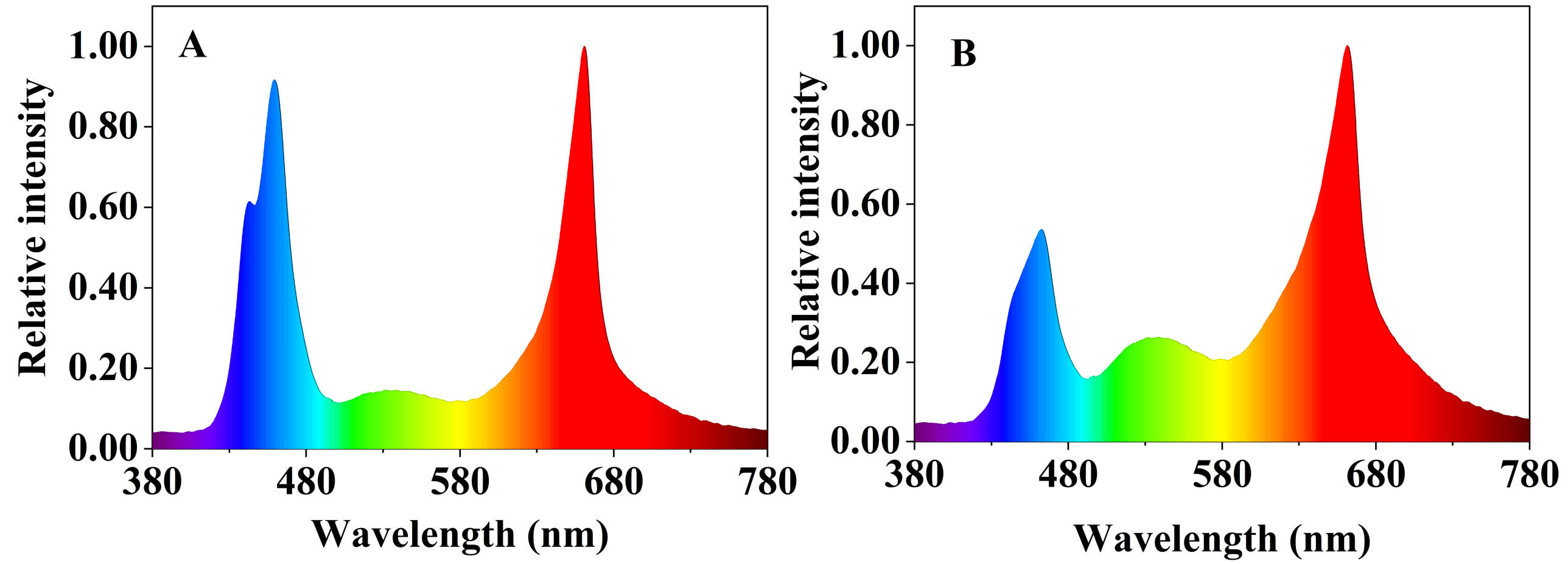

Supplement: Figure S1 [file peerj-10-14325-s002.png]
